# GPR110 promotes progression and metastasis of triple-negative breast cancer

**DOI:** 10.1038/s41420-022-01053-x

**Published:** 2022-05-26

**Authors:** Hye-Jung Nam, Yeon-Ju Kim, Jae-Hyeok Kang, Su-Jae Lee

**Affiliations:** Department of Life Science, Research Institute for Natural Sciences, Hanyang University, Seoul, Korea

**Keywords:** Breast cancer, Cell invasion

## Abstract

Breast cancer is the most common type of cancer in women, and approximately 70% of all breast cancer patients use endocrine therapy, such as estrogen receptor modulators and aromatase inhibitors. In particular, triple-negative breast cancer (TNBC) remains a major threat due to the lack of targeted treatment options and poor clinical outcomes. Here, we found that GPR110 was highly expressed in TNBC and GPR110 plays a key role in TNBC progression by engaging the RAS signaling pathway (via Gαs activation). High expression of GPR110 promoted EMT and CSC phenotypes in breast cancer. Consequently, our study highlights the critical role of GPR110 as a therapeutic target and inhibition of GPR110 could provide a therapeutic strategy for the treatment of TNBC patients.

## Introduction

Breast cancer is the most common type of malignant cancer in women [1] and can be divided into subtypes, Luminal, HER2+, and triple-negative breast cancer (TNBC) type. Specifically, TNBC is defined to be receptor non-dependent (estrogen, progesterone receptor, and human epidermal growth factor receptor 2). Also, TNBC is a relatively malignant type compared to other types and is difficult to treat [2]. For TNBC patients, the overall survival rate is remarkably low and indicates a high rate of relapse. Therefore, new therapeutic strategies for the treatment of TNBC are needed.

The RAS family includes the KRAS, HRAS, and NRAS subtypes. KRAS is the most frequent oncogene and accounts for 86% of the RAS family (HRAS and NRAS account for 3 and 11%, respectively) [3]. Extensive studies have shown that KRAS upregulation is associated with various malignancies, including lung adenocarcinoma, ductal carcinoma of the pancreas, colorectal carcinoma, and breast carcinoma [4, 5]. Hyperactivity and upregulation of KRAS are significantly associated with worse survival rates. Therefore, inhibition of KRAS activation is a popular and efficient method for cancer treatment [6].

The defining characteristic of adhesion G protein-coupled receptors (GPCRs) that discriminates them from other GPCRs is their hybrid molecular structure. Adhesion GPCRs contain an exceptionally long extracellular region and a variety of structural domains that facilitate cell and matrix interactions. The general signaling mechanism of GPCR begins by an interaction with the guanine nucleotide protein G protein (Gαs, Gαq, Gαs and Gα12/13) [7]. Several previous studies revealed that some members of the adhesion GPCRs can induce cancer progression by regulating angiogenesis, proliferation, metastasis, and survival through their signaling function [8–10]. A previous study showed that GPR110 accelerates liver fibrosis/cirrhosis progressing through activation of the IL-6/STAT3 pathway, leading to a liver injury and fibrosis microenvironment [11]. Furthermore, high expression of GPR110 predicts the poor prognosis of gastric cancer patients [12]. Despite these initial findings of GPR110, GPR110 are orphan receptors, and their signaling pathways have not been investigated in breast cancer. The mechanism and other physiological functions of GPR110 in cancer are also unclear.

In this study, we demonstrated that GPR110 promotes cancer progression by regulating EMT and cancer stem-like cell (CSC) property. Importantly, the increased level of GPR110 correlated with activation of the KRAS signaling pathway. Collectively, our results reveal that the oncogenic function of GPR110 is an important mechanism, resulting in metastasis and CSC in breast cancer, and suggest that GPR110 is a potential candidate for TNBC target therapy.

## Results

### GPR110 was highly expressed in TNBC

**Fig. 1:**
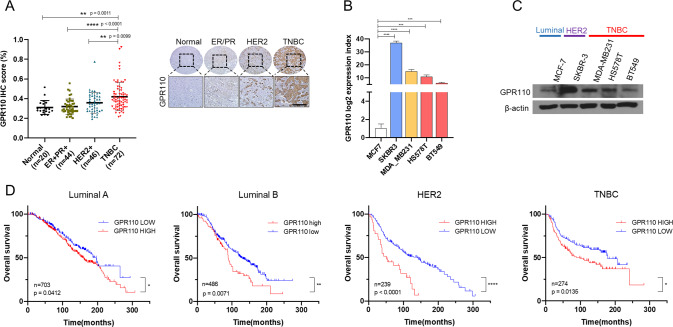
GPR110 was highly expressed in triple-negative breast cancer. **A** Representative IHC images of GPR110 staining in invasive ductal carcinoma (IDC) cancer tissue and the quantification graph. Image J software was used for analysis. **B**, **C** qRT-PCR analysis and Western blotting showed the expression of GRP110 in cell lines from the Luminal, Her2^+^, TNBC subtypes. **D** Kaplan–Meier survival analysis showed that high expression of GPR110 was associated with poor prognosis of Luminal A, Luminal B, Her2^+^, and TNBC type patients. *p < 0.05, **p < 0.001, ***p = 0.0001 and ****p < 0.0001; ns not significant; determined by two-tailed Student’s *t* test (95% confidence interval).

We performed The Cancer Genome Atlas Program (TCGA) database analysis and gene expression analysis to investigate total adhesion GPCR expression in luminal and basal breast cancer (Supplementary Fig. 1A, B). Among the two types of cancer, basal breast cancer was associated with GPR110 gene enrichment. Also, gene set enrichment analysis (GSEA) revealed that basal breast cancer-related signature gene sets were positively correlated with GPR110 expression levels. Although the expression of GPR116 or ELD1 was found to be more significant than GPR110, additional GEO database and TCGA analysis results showed no significance (Supplementary Fig. 1C–E). Therefore, we found that GPR110 had the most significant difference in expression in basal breast cancer compared to luminal breast cancer through GPCR expression analysis screening. Also, we assumed that the high expression of GPR110 in basal breast cancer would contribute to breast cancer malignancy. To confirm our data, we performed expression analysis of GPR110 using a breast scanner patient tissue array. The IHC staining revealed that GPR110 expression is significantly higher in TNBC patient tissues (Fig. 1A). Additionally, we performed quantitative reverse transcription-polymerase chain reaction (qRT-PCR) and western blotting analysis using different subtypes of breast cancer cell lines. The results showed that GPR110 expression was higher in the TNBC subtype of breast cancer (Fig. 1B, C). Kaplan–Meier survival analysis using the GEO database demonstrated that high levels of GPR110 were associated with a poor prognosis in patients with breast cancer (Fig. 1D). Overall, we found that GPR110 expression was higher in TNBC and was associated with poor prognosis in breast cancer patients.

### GPR110 is a key regulator of the epithelial–mesenchymal transition (EMT) in TNBC

**Fig. 2:**
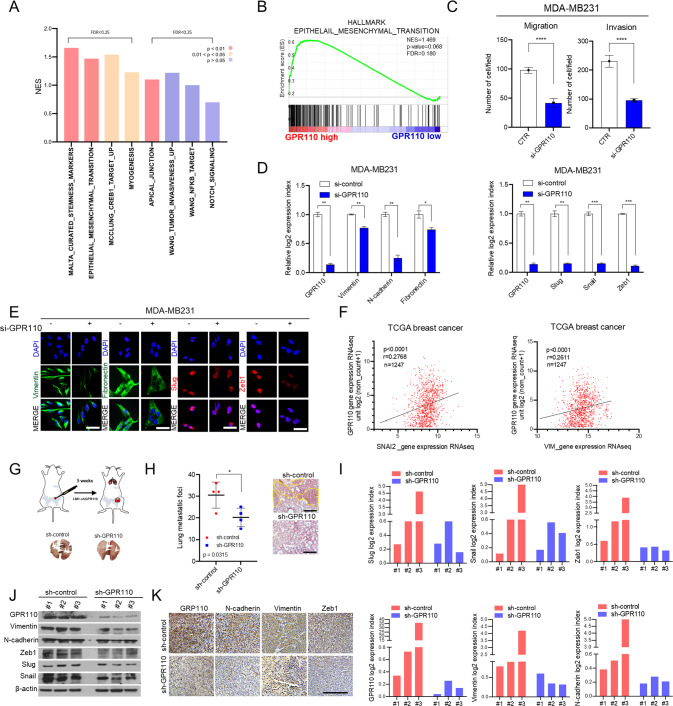
GPR110 is a key regulator of the epithelial–mesenchymal transition in breast cancer. **A**, **B** GSEA of hallmark epithelial-to-mesenchymal transition gene signature and stemness maker to GPR110 in breast cancer patients (GSE22516). **C** Invasion/migration assays were performed after silencing GPR110 in MDA-MB231 cells. **D** qRT-PCR analysis showing EMT markers and regulators after knockdown of GPR110 in MDA-MB231 cells. **E** Representative images of ICC staining of Vim, Fn, Slug, and Zeb1 in MDA-MB231 transfected with si-GPR110. **F** A positive correlation between Slug and GPR110 was observed using the public TCGA database (*n* = 1247). And a positive correlation between Vim and GPR110 was observed using the public TCGA database (*n* = 1247). **G**, **H** After injection into fat pad of female NOD/SCID mice, the image of mouse lungs and representative image of H&E staining of lung metastasis and the number of lung metastatic foci. **I**, **J** qRT-PCR analysis and Western blotting of EMT markers and regulators using mouse tissues. **K** IHC analysis of EMT markers and regulators in xenograft tumor of mice. Scale bar = 100 μm. β-actin was used as a control for normalization of expression. *p < 0.05, **p < 0.001, ***p = 0.0001 and ****p < 0.0001; ns not significant; determined by two-tailed Student’s *t* test (95% confidence interval).

To determine the effects of GPR110 and associated functional mechanisms, GSEA was performed. We found that EMT signature gene expression levels were positively correlated with GPR110 expression levels (Fig. 2A, B). GPR110 knockdown decreased the migration/invasion potential of cells and the expression of EMT markers (N-cadherin, fibronectin, and vimentin) and EMT regulators (Snail, Slug, Zeb1) (Fig. 2C, D). Similar results were obtained in the MDA-MB231cells by immunocytochemical (ICC) staining (Fig. 2E). Moreover, the expression levels of GPR110 and EMT signature gene (Slug or Vimentin) were positively correlated with each other in patients from the TCGA breast cancer clinical cohort (Fig. 2F). To validate our results in vivo, LM1 cells transfected with sh-GPR110 were injected into the fat pad of female NOD/SCID mice (Fig. 2G). In xenograft models, the number of lung metastatic foci and tumor weight were significantly lower in the sh-GPR110 group than in the control group (Fig. 1H). Notably, GPR110 inhibition decreased tumor growth (Fig. S2M, N). Western blotting and RT-qPCR analysis showed that suppression of GPR110 inhibited EMT signature genes and the proliferation marker Ki67 in primary tumor tissues (Fig. 2I, J and Fig. S2O). Immunohistochemical staining also revealed that the EMT signature was significantly muted in the sh-GPR110 group (Fig. 1K). Taken together, our data indicate that GPR110 upregulates EMT features in breast cancer.

### GPR110 promotes breast CSCs

**Fig. 3:**
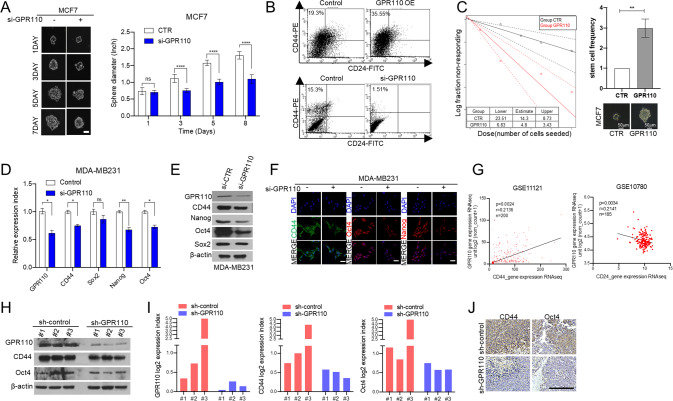
GPR110 promotes breast cancer stem-like cells. **A** Sphere formation assay was performed using GPR110-overexpressing MCF7 and the colony size was measured and shown in a graph. Representative images of forming cells showing the growth of the sphere (left). Scale bar: 20 μm. The graph showed the size of spheres formed (right). **B** Flow cytometric analysis of the percentage of CD44+/CD24− cells in the GPR110-overexpressing MCF7 and GPR110-silenced MDA-MB231 cells. **C** in vitro Limiting dilution assay was performed using GPR110-overexpressing MCF7 cells. **D**, **E** qRT-PCR and Western blotting analysis were performed to check the CSC regulators after silencing GPR110 in MDA-MB231. **F** Representative ICC Image of CD44, OCT4 and NANOG in GPR110-silenced MDA-MB231 cells. **G** A positive correlation between CD44 and GPR110 was observed using the public GSE database (GSE11121) and a negative correlation between CD24 and GPR110 was observed using the public GSE database (GSE10780). **H**–**J** Western blotting, qRT-PCR analysis, and IHC staining of CSC regulators using mouse tissues. Scale bar = 100 μm. *p < 0.05, **p < 0.001 and ****p < 0.0001; ns not significant; determined by two-tailed Student’s *t* test (95% confidence interval).

As previously shown in GSEA (Fig. 2A), it was found that CSC signature gene expression levels had a positive correlation with GPR110 expression. To investigate the molecular function of GPR110 for the CSC features, we performed a sphere formation assay in GPR110-silencing MCF7 cells. The results showed that the GPR110 knockdown group decreased the sphere diameter of a cell compared to the control group (Fig. 3A). Furthermore, FACS analysis showed that overexpressing of GPR110 increased the percentage of the stem cell population (CD44^+^/CD24^−^ cells) and silencing of GPR110 expression decreased the percentage of the stem cell population (CD44^+^/CD24^−^ cells) (Fig. 3B). In vitro limiting dilution assay analysis also showed that stem cell frequency was increased in overexpressing GPR110 cells compared to the control group (Fig. 3C). Subsequently, the expression of CSC regulators (CD44, Nanog, Oct4, and Sox2) was decreased in GPR110-silencing MDA-MB231 cells. (Fig. 3D–F). In addition, analysis of the GEO database for breast cancer patients showed a positive correlation between GPR110 expression and CD44 gene and a negative correlation between GPR110 expression and CD24 gene (Fig. 3G). Also, the sh-GPR110 group decreased the expression of CSC regulators compared to the sh-control group in xenograft tumors (Fig. 3H–J). Taken together, our data indicate that GPR110 upregulates CSC in breast cancer.

### GPR110 induces EMT and CSC features via Gas/RAS pathway in breast cancer

**Fig. 4:**
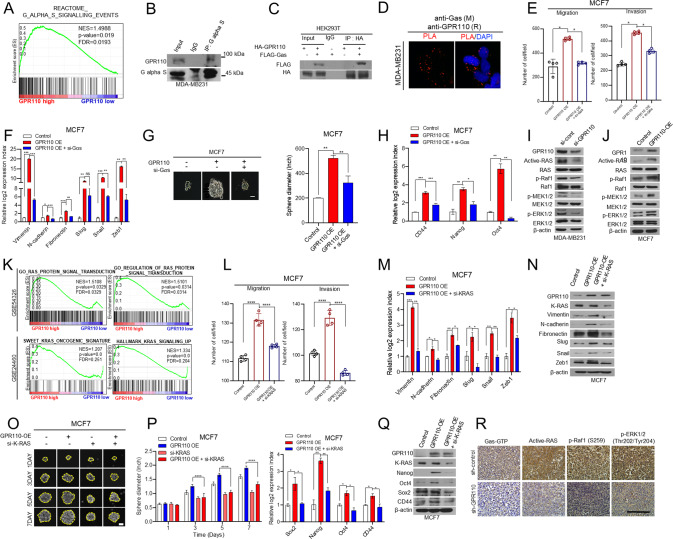
GPR110 induces epithelial–mesenchymal transition and cancer stem-like cells phenotype via Gαs/RAS pathway. **A** GESA analysis revealed that GPR110 expression was positively correlated with the Gαs signaling (GSEA12093). **B** Co-immunoprecipitation with Gas antibody and western blot analysis to check the interaction between Gas and GPR110 in MDA-MB231. **C** Co-Ip assay to analyze GPR110 and Gαs interaction in HEK293T cells. **D** Representative images and quantification of in situ PLA showing the interaction between Gas and GPR110. Scale bar = 100 μm. **E** The invasive and migrated cell numbers were assessed in GPR110 expression alone or together with Gαs expression in MCF7 cells. **F** qRT-PCR analysis of EMT markers and regulators using the same rescue experiments. **G** Sphere forming assay were assessed in GPR110 expression alone or together with Gαs expression in MCF7 cells. **H** qRT-PCR analysis of CSC regulators using the same rescue experiments condition. **I**, **J** Western blotting analysis to assess active RAS signaling pathway (p-RAF, p-MEK, p-ERK) using GPR11-silencing MDA-MB231 cells or GPR110-overexpressing MCF7 cells. **K** GSEA of RAS protein signal transduction signature and KRAS oncogene signature to GPR110 expression in breast cancer patients (GSE54326, GSE24460). **L** The invasive and migrated cell numbers were assessed in GPR110 expression alone or together with K-Ras expression in MCF7 cells. **M**, **N** Western blotting analysis and qRT-PCR analysis of EMT markers and regulators using the same rescue experiments condition. **O** Sphere forming assay was assessed in GPR110 expression alone or together with K-Ras expression in MCF7 cells (left) and the graph showed the size of spheres formed (right). **P**, **Q** Western blotting analysis and qRT-PCR analysis of CSC regulators using the same rescue experiments condition. **R** IHC analysis of GTP-Gas, Active-RAS, p-RAF, and p-ERK in xenograft tumor of mice. Scale bar = 100 μm. *p < 0.05, **p < 0.001, ***p = 0.0001 and ****p < 0.0001; ns not significant; determined by two-tailed Student’s *t* test (95% confidence interval).

As reported previously, adhesion GPCRs can activate an associated G protein [13]. The G protein contains subunit α together with a bound GTP, which can then dissociate from the β and γ subunits to further affect intracellular signaling proteins or target functional proteins directly depending on the α subunit type (G_α_s, G_α_q, G_α_q, and G_α12/13_). The G protein subunit acting on GPR110 is still not investigated in the mechanism of malignancy of breast cancer. Therefore, we performed western blotting to screen for G-proteins interacting with GPR110 in breast cancer cells. Results indicated that the inhibition of GPR110 decreased Gαs-GTP levels compared to that of the other subunit types (Fig. S4A). Furthermore, by performing GSEA, we found that expression of Gαs signaling signature genes was positively correlated with GPR110 expression (Fig. 4A). Immunoprecipitation analysis and in situ proximity ligation assays indicated that Gαs interacts with GPR110 (Fig. 4B–D). In addition, blocking Gαs alone or with GPR110 overexpression in MCF7 cells reversed increases in EMT/CSC-related gene expression, invasion/migration potential, and sphere size of colonies (Fig. 4E–H).

To investigate how GPR110 participates in the progression of malignant characteristics, we investigated the biological pathway that GPR110 uses to regulate EMT and CSC. In MDA-MB231 cell lines, we performed signaling pathway screening by inhibiting GPR110 and found that GPR110 regulated the activation of RAS, phospho-Raf1, phospho-MEK1/2, and phospho-ERK1/2 (Fig. S4D, I, J). GSEA analysis (GSE54326) also indicated that GPR110 levels were positively associated with the expression of signature genes in the RAS signaling pathway. Notably, GSEA analysis (GSE24460) showed that GPR110 positively correlated with KRAS signaling signature genes (Fig. 4K). Therefore, GPR110 can induce EMT and CSC by activating the KRAS pathway, suggesting that it can regulate KRAS hyperactivity that contributes to cancer progression by targeting GPR110. To further confirm these findings, we performed rescue experiments. GPR110 overexpression inhibited the invasive features of cells and EMT-related gene expression, while co-overexpression of GPR110 and KRAS reversed the inhibition. (Fig. 4L–N). Likewise, GPR110 overexpression suppressed the sphere-forming ability of cells and CSC-related gene expression, while overexpression of GPR110 and KRAS resulted in rescue (Fig. 4O–Q). Also, the sh-GPR110 group decreased the expression of RAS activity compared to the sh-control group in xenograft tumors (Fig. 4R). Taken together, these results demonstrate that GPR110 induces EMT and CSC features via Gas/RAS pathway.

### GPR110 indicated a poor prognosis of breast cancer

**Fig. 5:**
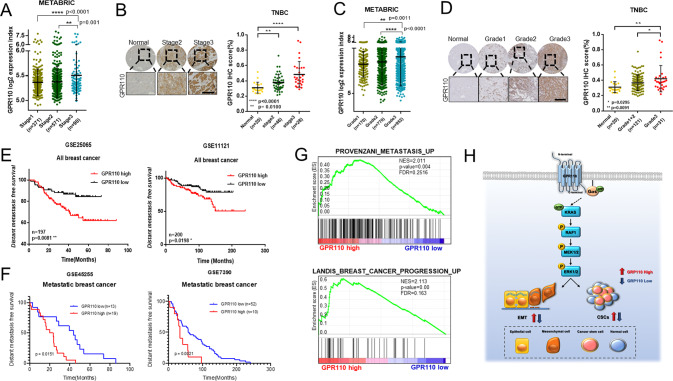
GPR110 indicated a poor prognosis of breast cancer. **A** Graph showing GPR110 expression in stage1 (*n* = 371), stage2 (*n* = 571), and stage3 (*n* = 90) of breast cancers using the database from METABRIC breast cancer databases. **B** Tissue microarray analysis of GPR110 expression in different stages of breast cancer (left) and the proportion is shown in the graph (right). **C** Graph showing GPR110 expression in grade1 (*n* = 170), grade2 (*n* = 770), and grade3 (*n* = 952) of breast cancers using the database from METABRIC breast cancer databases. **D** Tissue microarray analysis of GPR110 expression in different grades of breast cancer (left) and the proportion in shown in the graph (right). **E** Kaplan–Meier survival analysis showed that high expression of GPR110 was associated with a poor survival rate of breast cancer patients obtained from the GEO database (GSE25085, GSE11121). **F** Kapan–Meier survival analysis of metastatic breast cancer patients (GSE45255, GSE7390). **G** GSEA of metastasis and cancer progression signature to GPR110 expression in breast cancer patients. **H** Schematic of GPR110/Gαs/RAS signaling axis mechanism in breast cancer. *p < 0.05, **p < 0.001 and ****p < 0.0001; ns not significant; determined by two-tailed Student’s *t* test (95% confidence interval).

Together with the accumulated data, GPR110 was identified to be involved in EMT and CSC through the RAS pathway. This result shows that targeting GPR110 can lead to better survival of metastatic breast cancer patients by regulating cancer progression. Reanalysis of the METABRIC breast cancer database and immunohistochemical staining score also showed that overexpressed GPR110 expression was observed in high-stage and high-grade patients’ tissues (Fig. 5A–D). These contributions of GPR110 to the progression of cancer in GPR110 are associated with poor prognosis of all breast cancer patients, as obtained from Kaplan–Meier analysis of GSE25065 and GSE11121 (Fig. 5E). In addition, when comparing the survival of metastatic breast cancer patients, patients with high GPR110 expression showed a poor prognosis outcome (Fig. 5F). GSEA showed that patients with high GPR110 expression displayed a positive correlation with metastasis and breast cancer progression signature gene sets (Fig. 5G). Consequently, our study demonstrates that GPR110 plays a key role in TNBC progression by engaging the RAS signaling pathway (via Gαs activation) (Fig. 5H). Together, our study highlights the critical role of GPR110 as a therapeutic target. Inhibition of GPR110 could provide a therapeutic strategy for the treatment of TNBC patients.

## Discussion

Breast cancer is generally treated with surgery including chemotherapy of radiation therapy, or both. Also, hormone receptor-positive cancers are treated with hormone-blocking therapy and they can be identified by the presence of estrogen receptors and progesterone on their surface. ER+ cells depend on estrogen for their growth, so they can be treated with drugs such as tamoxifen or an aromatase inhibitor (e.g anastrozole). In addition, overexpression of HER2 in breast cancer is associated with disease recurrence and poor prognosis andHER2+ cells respond to monoclonal antibody trastuzumab and this has improved the prognosis significantly. However, TNBC does not have any of the three receptor types and differs from other types of invasive breast cancer in that they grow and spread faster and have limited treatment options. Outcomes of breast cancer vary depending on the cancer type, and the overall survival rate for TNBC patients is remarkably low.

G-protein coupled receptor 110 (GPR110) belongs to subfamily VI of adhesion GPCRs. In previous studies, It has been reported that GPR110 could promote the invasion and migration of lung and prostate cancer [14]. In addition, GPR110, which is highly expressed in an anti-HER2 therapy-resistant population [15], has a potential role in tumorigenesis. However, the clinical function, mechanism, and/or targets of GPR110 in breast cancer remain unknown.

In our research, we identified that GPR110 can interact with Gαs, resulting in the progression of breast cancer. First, we found that GPR110 is highly expressed in TNBC compared to other breast cancer subtypes, using human tissue array and GEO analysis. We also showed that the expression of GPR110 regulates EMT and CSC-like features to induce the metastatic phenotype of breast cancer by performing a GSEA public database analysis. In addition, we found that GPR110 can interact with Gαs through GSEA database analysis and co-immunoprecipitation assay. Therefore, we performed invasion/migration and sphere formation assays to identify the ability of GPR110/Gαs in breast cancer. The results showed that the cells’ ability to invade and migrate and form spheres rapidly increased according to the expression of GPR110 and activation of Gαs. These results reveal the mechanism by which GPR110 regulates EMT and CSCs through Gαs activation. Next, we found that GPR110 was associated with the RAS pathway and was especially correlated with KRAS from the GSEA database analysis. Furthermore, western blotting analysis, RT-qPCR analysis, and animal experiments showed that GPR110 could upregulate RAS activity and its downstream effector activation, such as the Raf and ERK signaling pathways. The results showed that GPR110 has the potential to control EMT and CSCs through active KRAS. Rescue experiments showed that when GPR110 was overexpressed in MCF-7 cells, which represent a luminal type of breast cancer, the expression of EMT and CSC markers and regulators increased, while their expression was inhibited when KRAS expression was knocked down in GPR110-overexpressing MCF-7 cells. Consequently, we found that GPR110 promoted EMT and CSCs through the Gαs-RAS signaling pathway in breast cancer. However, this study has some limitations. The upstream regulator of GPR110 is still unclear. Also, we do not know whether this occurs in other human malignancies. Further studies are required to address these limitations.

In summary, our findings demonstrate the importance of GPR110 in metastatic features and cancer formation in TNBC. We found that GPR110 expression was higher in TNBC compared to luminal breast cancer and that a high expression of GRP110 correlates with poor outcomes. In addition, we found that GPR110 activates KRAS and regulates EMT and CSC in TNBC. In contrast, loss of GPR110 function results in EMT and CSC attenuation and a better outcome prognosis in breast cancer patients. Taken together, targeting the GPR110/Gαs/RAS signaling pathway axis provides an effective strategy and mechanical system to relieve malignancies and improve the poor prognosis of patients with TNBC.

## Materials and methods

### Cell culture

MDA-MB231, HS578T, BT549, SKBR-3, and MCF7 breast cells were obtained from KCLB (Korean cell line bank). HECK29T cells were obtained from American type culture collection (ATCC). MDA-MB231, HS578T, LM1, and HECK293T cell lines were maintained in Dulbecco’s modified Eagle’s medium with 10% fetal bovine serum (FBS) in the presence of penicillin/streptomycin. MCF7, SKBR3, and BT549 cell lines were maintained in Roswell Park Memorial institute 1640 medium from Gibco (Grand Island, NY, USA) with 10% FBS in presence of penicillin (100 U/mL)/streptomycin (100 μg/mL).

### Chemical reagents and antibodies

Antibodies to pan-RAS (ab52939), H-RAS (ab97488), N-RAS (ab77392), K-RAS (ab137739), Raf1 (ab137435), p-JAK1 (sc-101716), p-ERK1/2 (sc-7383), AKT (sc-5298), STAT3 (sc-482), JAK1 (sc-7228), SRC (sc-8056), OCT4 (sc-9081), SOX2 (ab97959) and β-actin (sc-47778), anti-mouse IgG-HRP, anti-goat IgG-HRP, and anti-rabbit Ig-HRP were purchased from Santa Cruz Biotechnology (Santa Cruz, CA, USA). Antibodies to Anti-Goat Alexa Fluor 488, and anti-mouse Alexa Fluor 546 were purchased from Invitrogen (Carlsbad, CA, USA). Antibodies to ERK (4695), CD44 (ab157107), Nanog (ab21624), Slug (ab27568), Zeb1 (ab124512), Vimentin (ab8978), and Fibronectin (ab6329) were purchased from Abcam (Cambridge, UK). Antibodies MEK1/2 (8727), p-MEK1/2 (9121), p-JNK (9251), JNK (9252), p-Src (9211S), p-AKT (4060), Snail (3879S), P38 (9212), p-P38 (9211S) and p-STAT3 (9131) were purchased from Cell Signaling Technology (Beverly, MA, USA). Antibodies to GPR110 were purchased from LSbio. Antibodies to Active-RAS were purchased from NewEast, Antibodies to p-Raf1 were purchased from MyBioSource. Antibodies to Ki-67 were purchased from Actis. Antibodies to N-cadherin and β-catenin were purchased from BD biosciences. Vector of GPR110 were purchased from origene (ADGRF1, NM_153840, RC222706).

### Western blot analysis

Cell lysates were prepared by extracting proteins with lysis buffer [40 mM Tris–HCl (pH 8.0), 120 mM NaCl, 0.1% Nonidet-P40] which was supplemented with protease inhibitors. Proteins were separated by sodium dodecyl sulfate-polyacrylamide gel electrophoresis and transferred to a nitrocellulose membrane (Amersham, Arlington Heights, IL). Blocking the membrane with 5% non-fat dry milk in Tris-buffered saline, and incubated with primary antibodies overnight at 4 °C. Blots were developed with a peroxidase-conjugated secondary antibody, and proteins were visualized by enhanced chemiluminescence (ECL) procedures (Amersham, Arlington Heights, IL), using the manufacturer’s protocol.

### RNA preparation and real-time quantitative-PCR

Total RNA was isolated using Trizol reagent (Invitrogen, Carlsbad, CA, USA). All qRT-PCR was performed using the KAPA SYBR FAST qPCR kit from KAPA Biosystems (Wilmington, MA, USA), according to the manufacturer’s instructions. Reactions were carried out in the Rotor-Gene Q system (QUIGEN, Seoul, Korea), and results were expressed as fold change calculated by the ΔΔCt method relative to the control sample. β-actin was used as an internal normalization control for each sample. Beta-actin served as an internal normalization control. All primers were purchased from Macrogen (Seoul, Korea).

### Cell migration and invasion assays

For invasion assay, breast cancer cells were loaded in Transwells with 8 μm pore size filter inserts (Corning Glass, Seoul, Korea) that were precoated with 10 mg/mL growth factor-reduced matrigel (BD Biosciences, Seoul, Korea) on the upper side of the chamber with the lower well filled with 0.8 ml of growth medium. After incubation for 48 h at 37 °C, and For migration assay, we used Transwells with inserts that contained the same type of membrane but without the matrigel coating. After incubation for 24 h at 37 °C. Non-invaded cells on the upper surface of the filter were removed with a cotton swab and migrated cells on the lower surface of the filter were fixed and stained with the Diff-Quick kit (Fisher, Pittsburgh, PA, USA) and photographed (magnification ×20). Invasiveness and motility were determined by counting cells in four microscopic fields per well, and the extent of invasion was expressed as an average number of cells per microscopic field. Cells were imaged by phase-contrast microscopy (Leica Microsystems, Bannockburn, IL). All experiments were repeated three times.

### Immunocytochemistry

For immunoprecipitation assay, cells were fixed with 4% paraformaldehyde and permeabilized with 0.1% Triton X-100 in phosphate-buffered saline (PBS). and cells were blocked with 10% FBS and with 10% NP-40. Following fixation and blocking, cells were incubated at 4 °C overnight with the primary antibody in PBS with 1% bovine serum albumin (BSA) and 0.1% Triton X-100. Cells were visualized using anti-Rabbit or anti-Mouse Alexa Flour 488 or 546 (Molecular Probes, Seoul, Korea) Nuclei were counterstained with DAPI (Sigma). Stained cells were observed with a fluorescence microscope (Olympus IX71).

### Transfection

Cells were transfected with siRNA and/or overexpression vector using Lipopectamine 2000 (Invitrogen) or polyethylenimine (PEI) according to the manufacturer’s instructions. After 48 h transfection, cells were harvested. All siRNA was purchased from Genolution Pharmaceuticals, Inc. (Seoul, Korea). All experiments were independently repeated three times with similar results.

### IHC analysis

Mice were sacrificed and mice tissues were fixed in formalin for the preparation of paraffin sections. Paraffin-embedded tissue sections were deparaffinized in xylene, 100%, 95%, 80%, and 70% ethanol, followed by PBS. Epitopes were unmasked with 20 mg/mL proteinase K in PBS with 0.1% Triton X-100. Sections were stained with Hematoxylin and eosin (H&E) or immunostained overnight at 4 °C with the primary antibody in a blocking buffer with 5% Normal Goat Serum (NSG) and 3% BSA (BSA) in PBS. After washing in PBS, biotinylated goat anti-rabbit IgG or anti-mouse IgG antibody was then applied to the sections for 1 h. After washing in PBS, ABC reagent (Vector Laboratories Inc, Burlingame, CA, USA) was applied to the sections for 1 h. The color reaction was performed with 3,3’-diaminobenzidine (Vector Laboratories, Burlingame, CA, USA). After counter-staining with hematoxylin and clearing with graded ethanol series and xylene, the sections were mounted with Canada balsam. Images were captured with a DP71 digital imaging system on an IX71 microscope (Olympus, Seoul, Korea).

### Mouse experiments

All animal experiments were performed according to the guidelines of the Institutional Animal Care and Use Committee of Academia Sinica. LM1 control cells or LM1 sh-GPR110 cells (1 × 10^6^) suspended in 40 μL PBS were injected into the fat pad of 7–10-week-old female NOD/SCID mice (*n* = 8). Mice were anesthetized and a small incision was made to expose the mammary gland. Mice with tumors of representative size and weight in each group were sacrificed at day 32 after implantation. Primary mammary tumor growth was measured in 5-day intervals after injection. Tumor volumes were determined by measuring the length (I) and width (w), and the following formula was used for calculation: (shortest diameter^2^ × longest diameter/2). Lung metastatic foci were also counted after sacrifice.

### Human tissue microarray

Human breast cancer tissue microarray samples were obtained from US-Biomax (BR20838). These samples were reviewed by a pathologist to confirm the diagnosis of breast carcinoma and histological grade. Images were captured with a DP71 digital imaging system on an IX71 microscope (Olympus, Seoul, Korea).

### Proliferation assay

Cell proliferation efficiency was measured using the Cell Counting Kit-8 (CCK-8, KTA1020, Abbkine) according to the manufacturer’s instructions. Cells (5000 cells/well) were plated in 96-well plates. Afterward, the plates were incubated for an appropriate length of time (24, 48, 72, or 96 h), and 10 μl of CCK-8 solution was added to each well of the plate. The plate was incubated for 3 h at 37 °C in an incubator. The absorbance at 450 nm was measured using a microplate reader.

### Sphere formation assay

MCF-7cells (1.5 × 10^5^) were incubated for 7 days at 37 °C in an incubator. For the sphere-forming assay, the size of spheres was monitored on days 1–7 using the Motic Images Plus 2.0 software in six randomly chosen fields. The size of each randomly taken sphere was calculated as the average value and the error value of the entire sphere. Quantification was performed using the Image J software.

### Limiting dilution assay

Transfected cells were plated in 96-well plates at concentrations of 1, 2, 5, and 10 cells per well. Plates were analyzed by light microscopy for oncospheres, 10–14 days after plating. Positive wells were defined as groups of cells >125 μm in diameter.

### Flow cytometric analysis

Flow cytometry was used to detect the CSC markers CD44 and CD24. A total of 1 × 10^6^ control cells and GPR110-overexpressing cells were harvested by trypsin digestion, washed, and resuspended in 1× PBS. The cells were incubated with R-phycoerythrin (PE)-conjugated anti-CD44 monoclonal antibody and FITC-conjugated anti-CD24 antibody (Miltenyi Biotec, Inc., Bergisch Gladbach, Germany) at 4 °C for 30 min. All data were analyzed using the CellQuest software (BD Biosciences) and repeated three times.

### GSEA, data set evaluation, and Kaplan–Meier analysis

GSEA was performed on diverse gene signatures by comparing gene sets from either the Molecular Signature Database (MSigDB) or published gene signatures. To analyze the expression of GPR110 in breast cancer, previously published microarray data under accession codes GSE20685, GSE5327, GSE58812, GSE14548, GSE12777, GSE21653, GSE2034, GSE11121, GSE5364, GSE54326, GSE24460, GSE22516, GSE7904, GSE12276. TCGA and METABRIC database mRNA expression *z*-score data sets along the PAM50 gene set were retrieved from UCSC Xena (https://xenabrowser.net/heatmap/) were reanalyzed. To examine the prognostic value of GPR110, patient samples were divided into two groups (low and high expression) for each gene, which was analyzed using the Kaplan–Meier plot program (http://kmplot.com/analysis/) and Breast Cancer Integrative platform (BCIP) program (http://www.omicsnet.org/bcancer/database).

### Statistical analysis

All experimental data are presented as the mean ± SD of triplication. Statistical analyses were performed using Student’s *t* test. Multiple group comparisons were made by analysis of variance using the PRISM 8.0 software (GraphPad). The level of significance is indicated as *p* < 0.05*.

## Supplementary information

Supplementary file

## Data Availability

The data supporting the finding of this study are available from the corresponding author upon reasonable request.
