# Correction: GPR110 promotes progression and metastasis of triple-negative breast cancer

**DOI:** 10.1038/s41420-022-01266-0

**Published:** 2022-12-07

**Authors:** Hye-Jung Nam, Yeon-Ju Kim, Jae-Hyeok Kang, Su-Jae Lee

**Affiliations:** Department of Life Science, Research Institute for Natural Sciences, Hanyang University, Seoul, Korea

**Keywords:** Breast cancer, Cell invasion

Correction to: *Cell Death Discovery* 10.1038/s41420-022-01053-x, published online 26 May 2022

The original version of this article contained an error. The authors found that the number was entered incorrectly in the funding part of the written article. “2020R1A4A101839812” should be modified to “2020R1A4A1018398”. The authors apologize for the error. The original article has been corrected.

