# Supplementary material for: GPR110 promotes progression and metastasis of triple-negative breast cancer
Source: Cell Death Discov. 2022 May 26;8:271. doi: 10.1038/s41420-022-01053-x (PMC9132940; doi:10.1038/s41420-022-01053-x)
Supplement: Supplementary file 1 — Supplementary file [file 41420_2022_1053_MOESM1_ESM.docx]

**
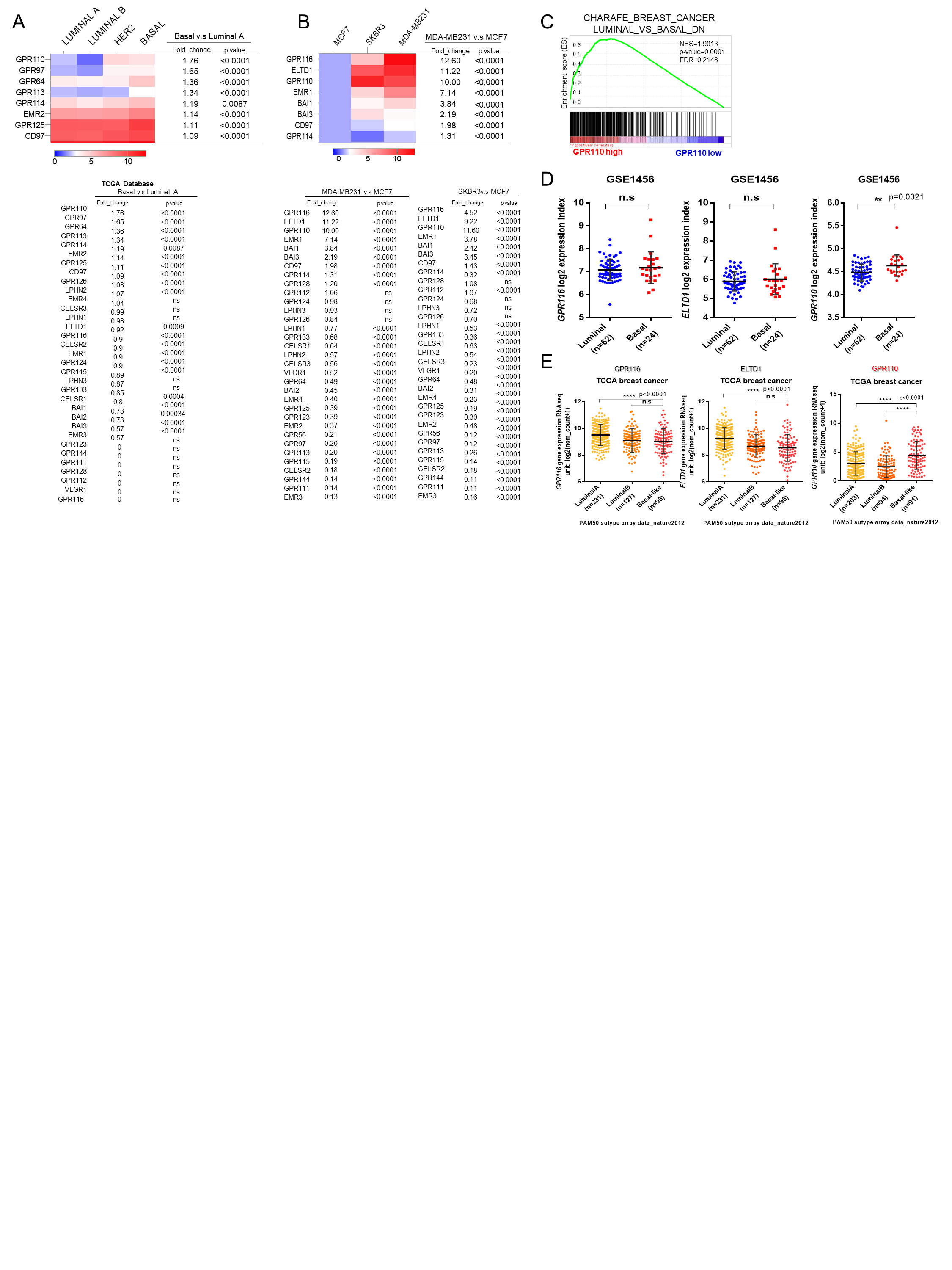
**

**Fig. S1. GPR110 was high expressed in triple negative breast cancer**

(A) Heatmap showed the ranked list of adhesion GPCR expressed high level in Luminal A, Luminal B, Her2+ and Basal type defined by PAM50 expression from RNA-seq data using the data from the TCGA breast cancer database, respectively. (B) Heatmap showed the ranked list of adhesion GPCR expressed high level in TNBC type, MDA-MB231 cell by fold change in descending order obtained by qRT-PCR analysis. Red and blue indicate upregulation and downregulation. The fold change calculated by log^2^ expression of basal divided by log^2^ expression of luminal type breast cancer. (C) GSEA of luminal versus basal gene signature of breast cancer from GSE22516. (D-E) analysis expression of Top 3 GPCR (GPR116, ELTD1 and GPR110) in the patients with the luminal and basal subtypes of breast cancer using GSE database (GSE1456) and TCGA. *p < 0.05, **p < 0.001, and ***p < 0.0001; ns, not significant; determined by two-tailed Student’s t-test (95% confidence interval).


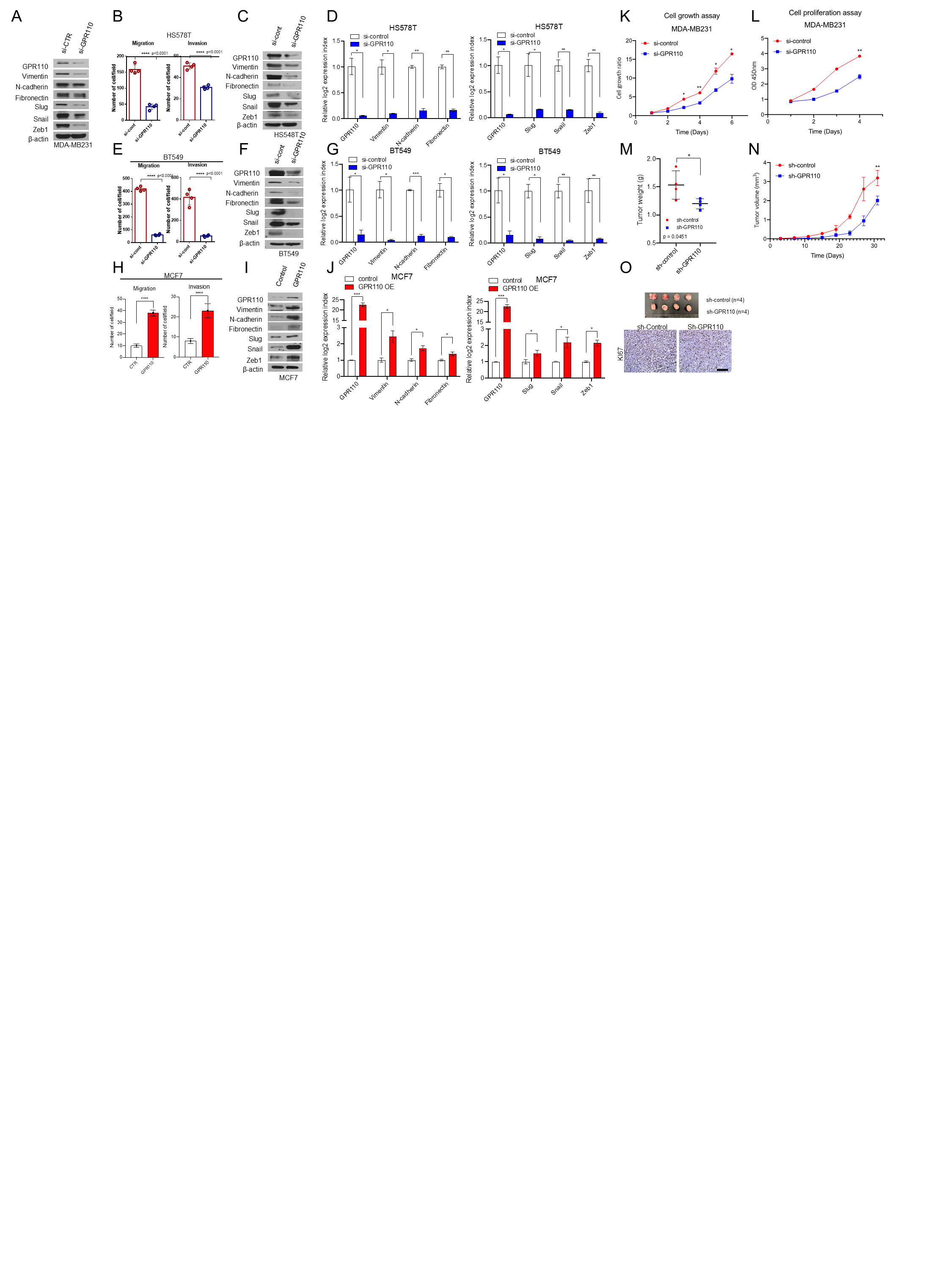


**Fig. S2 GPR110 is a key regulator of the epithelial-mesenchymal transition in breast cancer**

(A) Western blotting analysis were performed to check EMT marker and regulator after silencing GPR110 in MDA-MB231 cells. (B-G) The invasive and migrated cell numbers were assessed using knockdown of GPR110 in HS578T or BT549cells. qRT-PCR and Western blotting analysis showing EMT markers and regulators were assessed using knockdown of GPR110 in HS578T or BT549 cells. (H-J) The invasive and migrated cell numbers were assessed using overexpression of GPR110 in MCF7 cells. qRT-PCR and Western blotting analysis showing EMT markers and regulators were assessed using overexpression of GPR110 in MCF7 cells. (K-L) cell growth assay of the si-control and si-GPR110 in MDA-MB231 cells. This graph displays results of cell counting for 7 days period. l. Cell proliferation assay results showing that the decrease in cell proliferation in MDA-MB231 cells by GPR110 knockdown. (M) The graph showed tumor weights from the control and GPR110-overexpressed xenograft group (n=5 for each group). (N)After injection into fatpad of female NOD/SCID mice, the volume of the tumor measures every five days for 5 weeks. (O) primary tumor images and representative Ki67 staining in primary tumor tissues. *p < 0.05, **p < 0.001, and ***p < 0.0001; ns, not significant; determined by two-tailed Student’s t-test (95% confidence interval).

**
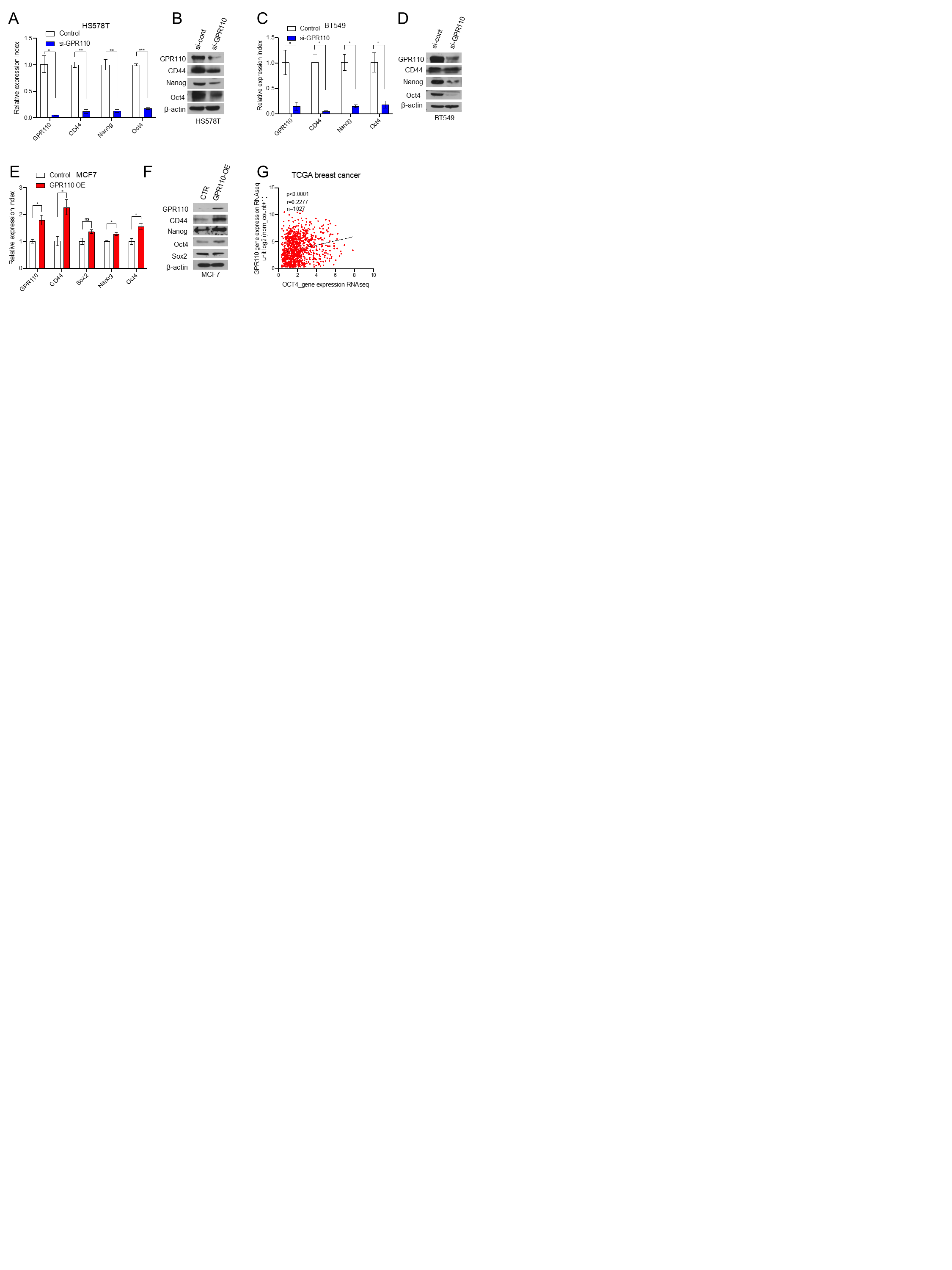
**

**Fig. S3 GPR110 promotes breast cancer stem-like cells**

(A-D) Western blotting and qRT-PCR analysis of CSC regulators after knockdown of GPR110 in HS578T or BT549 cells, respectively. (E-F) Western blotting and qRT-PCR analysis of CSC regulators after overexpression of GPR110 in MCF7 cells, respectively. (G) A positive correlation between OCT4 and GPR110 was observed using the public TCGA database. β-actin was used as a control for normalization of expression. *p < 0.05, **p < 0.001, and ***p < 0.0001; ns, not significant; determined by two-tailed Student’s t-test (95% confidence interval).


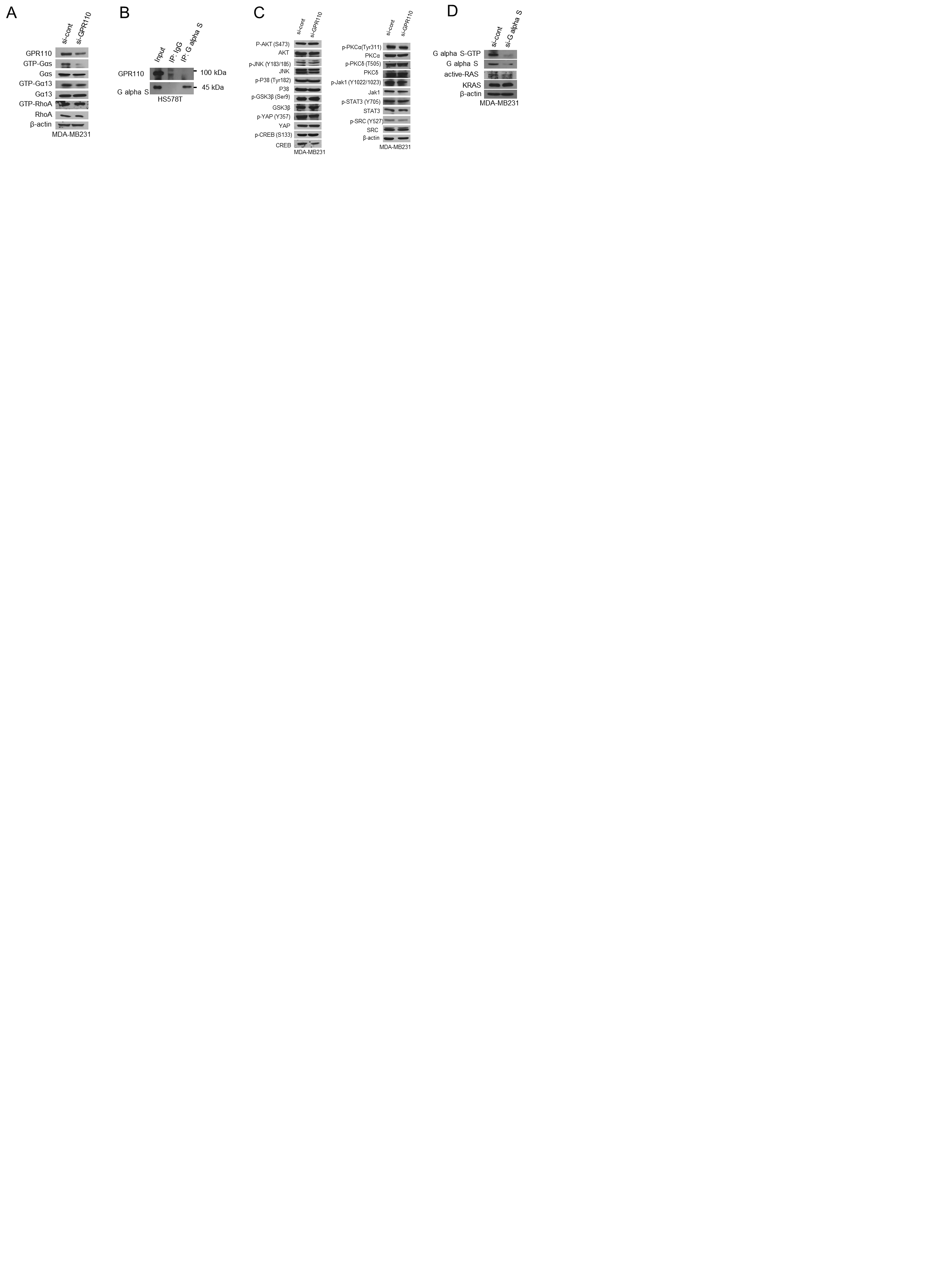


**Fig. S4 GPR110 induces epithelial-mesenchymal transition and cancer stem-like cells phenotype via Gαs/RAS pathway**

(A) Western blot analysis for the screening of G protein in GPR110-silenced MDA-MB231 cells. (B) Co-Ip assay to analysis GPR110 and Gαs interaction in HS578T cells. (C) The biological pathway screening using the western blotting analysis in GPR110-silenced MDA-MB231 cells. (D) Western blotting analysis for the K-Ras activity in Gαs-silenced MDA-MB231 cells. *p < 0.05, **p < 0.001, and ***p < 0.0001; ns, not significant; determined by two-tailed Student’s t-test (95% confidence interval).
